# Two novel mutations identified in two Chinese gelatinous drop-like corneal dystrophy families

**Published:** 2007-06-24

**Authors:** Bei Zhang, Yu-Feng Yao, Ping Zhou

**Affiliations:** Department of Ophthalmology, Affiliated Sir Run Run Shaw Hospital, Zhejiang University School of Medicine, Zhejiang, P. R. China

## Abstract

**Purpose:**

To identify the genetic defect in the *TACSTD2* gene that causes gelatinous drop-like corneal dystrophy (GDLD) in two unrelated consanguineous Chinese families.

**Methods:**

Genomic DNA was prepared from leukocytes of peripheral venous blood. The coding region of the *TACSTD2* gene was evaluated by means of polymerase chain reaction and direct sequencing.

**Results:**

Sequencing of the *TACSTD2* gene of the two probands revealed two novel homozygous frameshift mutations: c.84insG and c.480delC. The identified molecular defect cosegregates with the disease among affected members of the families and is not found in 50 unaffected controls.

**Conclusions:**

This study reports two novel mutations in two GDLD families and expands the spectrum of mutations in *TACSTD2* gene that may cause pathological corneal amyloidosis.

## Introduction

Gelatinous drop-like corneal dystrophy (GDLD; OMIM 204870) is a rare autosomal recessive corneal dystrophy, mostly reported in the Japanese population, with an estimated prevalence of 1 in 33,000 [1]. Clinical manifestations usually appear in the first decade of life with bilateral, axial, elevated, mulberry-like gelatinous lesions, due to primary amyloid deposition in the subepithelium and anterior stroma of the cornea. The amyloid deposits in the cornea will progressively spread laterally and deeply within the stroma, leading to a progressive opacification of the cornea and resulting in severe visual damage, photophobia, and foreign-body sensation. In the severe cases, corneal transplantation is required for recovery of corneal clarity and visual rehabilitation [2,3].

Conventional position cloning revealed that the gene responsible for most cases of GDLD was tumor-associated calcium signal transducer 2 (*TACSTD2*, formerly *Trop2*, *GA733-1*, and *M1S1*) located at 1p32 [4]. The *TACSTD2* gene consists of a single exon spanning about 1.8 kb of genomic DNA. It codes for a protein of 323 amino acids, which is a monomeric cell surface glycoprotein expressed in the cornea, multistratified epithelia, and trophoblasts and is found in most carcinomas with high protein expression levels [4,5]. The physiological function of *TACSTD2* has not been exactly elucidated, whereas it is hypothesized that protein may act as a calcium signal transducer [5].

Four mutations causing GDLD have been found in the *TACSTD2* gene in Japanese patients: Q118X, Q207X, S170X, and c.632delA [4]. All four mutations generate an early stop codon and lead to synthesis of a truncated protein. Molecular genetic analysis of patients from diverse ethnic backgrounds showed both genetic and allelic heterogeneity for GDLD [6-10].

In the present study we report the molecular genetic analysis of *TACSTD2* in two unrelated consanguineous families with GDLD, in which two novel *TACSTD2* mutations were identified. To our knowledge this is the first report of GDLD with novel gene mutations found in families from the southern and southeastern region of China.

## Methods

### Subjects

**Figure 1:**
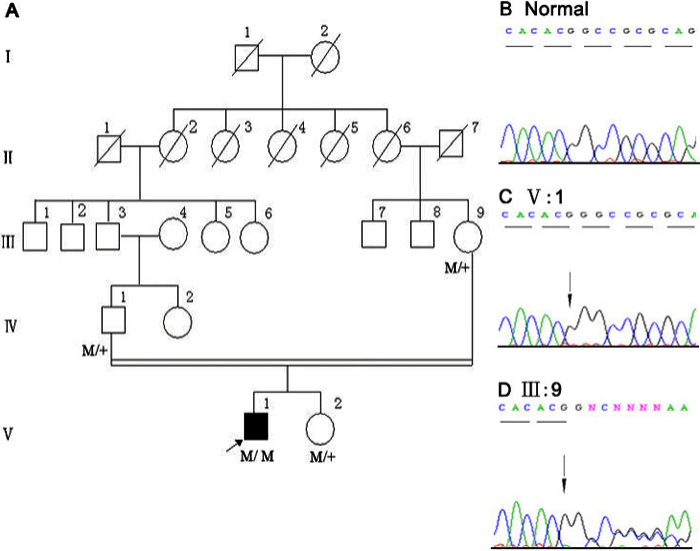
Novel *TACSTD2* mutation in family 1. **A**: In this pedigree of the study family, M represents the mutant allele and + indicates the wild-type allele. The proband is marked by an arrow. In the family tree, squares indicate male and circles indicate female of the family members. Slashes denote family members who were deceased, whereas heavy shading means the individual who was affected by GDLD. Double lines represent consanguineous spouses. **B**: Sequence analysis of *TACSTD2* gene near codon 28 detected in a healthy control. **C**: The sequence from proband A shows a homozygous insertion mutation, c.84insG (arrow). **D**: Double-wave peaks are seen after codon 28 (arrow) resulting from nonmatching of nucleotide sequence in two alleles of family member II:9.

**Figure 2:**
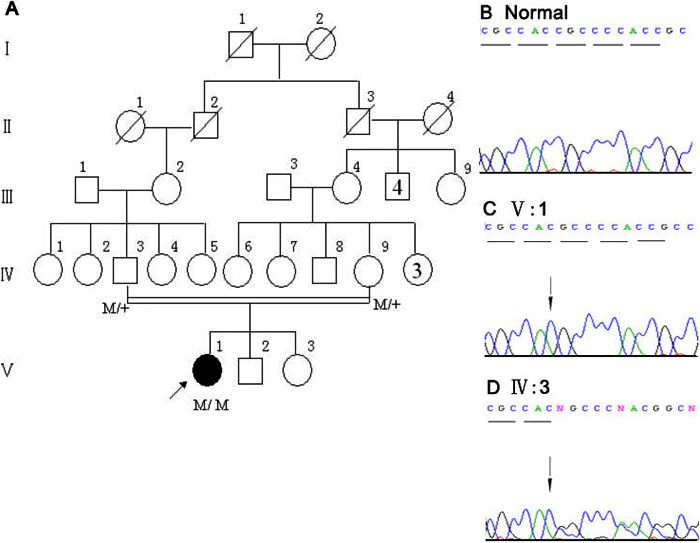
Novel *TACSTD2* mutation in family 2. **A**: Pedigree of the study family. In the family tree, squares indicate male and circles indicate female of the family members. Slashes denote family members who were deceased, whereas heavy shading means the individual who was affected by GDLD. Double lines represent consanguineous spouses. The numbers in the symbol in generation II and IV designate the number of siblings. **B**: Normal sequence of *TACSTD2* gene near codon 160. **C**: The sequence from proband B shows a deletion of C, c.480delC (arrow). **D**: Double-wave peaks are seen after codon160 (arrow) resulting from nonmatching of nucleotide sequence in two alleles of family member IV:3.

Two unrelated Chinese families with GDLD were examined. Family 1, consisting of 13 living individuals, 7 males and 6 females, with age range from 16 to 72 years, had only one member (patient V:1) affected by GDLD. Family 2 involved 24 living individuals, including 9 males and 15 females, with age range from 15 to 76 years, in which one of the family members (patient V:1) affected by GDLD. All participants received a detailed clinical examination. The probands and all available family members were enrolled in the study (Figure 1A, Figure 2A). Diagnosis of GDLD was based on characteristic clinical manifestations of the corneas in two patients and was further confirmed by histopathologic examination of the corneal tissues from the patients who received lamellar keratoplasty.

The study was approved by the institutional ethics committee of Sir Run Run Shaw Hospital. Informed consent was obtained from the patients and their family members who participated in this study.

### Mutation Analysis

Molecular genetic analyses were performed in the two probands and unaffected family members: members III:9, IV:1, and V:2 from family 1 and members IV:3 and IV:9 from family 2 (Figure 1A, Figure 2A). Fifty healthy Chinese subjects were examined as controls. Genomic DNA was extracted from peripheral blood by standard procedures [11]. The single exon of the *TACSTD2* gene was amplified using three sets of primers to generate overlapping products, which were screened for mutations by direct sequencing. The primers used in this study were identical to those reported by Akhtar et al. [12]. Their sequences are as follows: TACSTD2; F1/R1, 5'-ACG TGT CCC ACC AAC AAG AT-3' / 5'-CAG GTA ATA GAT GAG CGT GCG-3', TACSTD2; F2/R2, 5'-GGA TGT GTC ACC CAA ATA CCA-3' / 5'-CTT GAG CAG CAG ACA CTT GGA -3', and TACSTD2; F3/R3, 5'-CCT ACT ACT TCG AGA GGG ACA -3' / 5'-CAG GAA GCG TGA CTC ACT T-3'. The sizes of the amplified DNA fragments are 681 bp, 423 bp, and 382 bp, respectively.

Polymerase chain reaction (PCR) was carried out in a volume of 50 μl mixture containing 1 μM of each primer, 0.5 U of Hotstar Taq polymerase (Qiagen, Hilden, Germany), 250 μM dNTP mixture, 5 μl of 10X PCR buffer with MgCl_2_, 10 μl of 5X Q-solution (Qiagen), and approximately 100 ng of human genomic DNA. Thermal cycling was performed using a GeneAmp PCR System 9700 (Applied Biosystems, Foster City, CA) with the following program: 15 min at 95 °C, followed by 32 cycles of 94 °C for 1 min, 60 °C for 1 min, and 72 °C for 1 min, with a final extension step at 72 °C for 7 min.

Amplified DNA was purified using the QIAquick PCR purification kit (Qiagen) and sequenced according to the protocols accompanying the BigDye Terminator cycle sequencing kit (Applied Biosystems). An ABI Prism 377 Genetic Analyser (Applied Biosystems) was used to collect and analyze the sequence data. DNA was bidirectionally sequenced.

## Results

### Clinical findings

**Figure 3:**
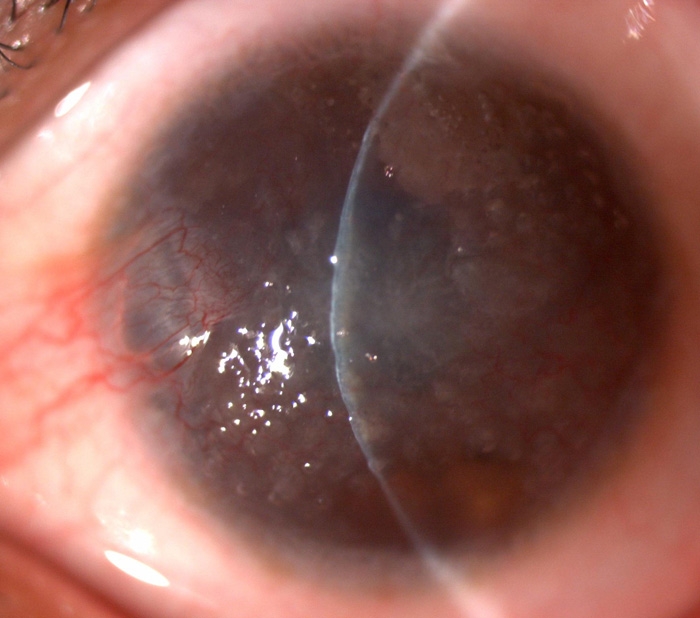
Slitlamp photograph of the left cornea of proband A. Shown is a diffuse corneal opacity with multiple grayish-white nodular elevations, almost from limbus to limbus, accompanied by neovascularization.

Proband A (V:1 from family 1) was a 16-year-old boy, born of a normal pregnancy to a consanguineous family in Zhejiang province in the southeastern region of China. Beginning at the age of 7, he experienced a slowly progressive vision loss accompanied with photophobia and lacrimation of both eyes. His visual acuity was limited to hand movement at 1 m in both eyes at the time of presentation. Examination revealed bilateral diffuse corneal opacities with multiple grayish-white nodular elevations located in the subepithelial area (Figure 3). The corneal opacification was symmetric in both eyes. The patient had no significant systemic diseases. Ophthalmic examination of his parents and sister did not reveal any ocular abnormalities, and there were no known similar familial ocular conditions according to the report from other family members.

Proband B (V:1 from family 2) was a 15-year-old girl from a consanguineous family in Yunan province in southern China. She presented with the same symptoms as proband A. Family history was negative. Corneal examination revealed less numerous small gelatinous opacities in the subepithelial area than proband A. The lesions were mainly in the central cornea with no vascularization in both corneas, and the peripheral cornea and deeper layers of the cornea were unremarkable. Best-corrected visual acuity was 10/60 OD and 20/80 OS. Ophthalmic examination of his parents did not reveal any ocular abnormalities.

### Molecular Analysis

Analysis of the sequence data from proband A revealed a homozygous mutation, c.84insG in *TACSTD2* (Figure 1C), resulting in a frameshift after codon 28 and a predicted stop codon at nt280. This mutation was confirmed by analysis of the reverse sequence data. Heterozygous mutations were detected in unaffected family members IV:1, III:9, and V:2. Double-wave peaks were seen after codon 28, resulting from nonmatching of nucleotide sequence in two alleles (Figure 1D). No mutations were detected in healthy controls (Figure 1B).

DNA analysis identified a homozygous mutation in *TACSTD2* at codon160 (Figure 2C) in proband B. The mutation was a single-base deletion, c.480delC, resulting in a frameshift mutation and a predicted stop codon at nt526. This mutation was confirmed by analysis of the reverse sequence data and was heterozygous in her parent (Figure 2D). No mutations were detected in healthy controls (Figure 2B).

## Discussion

GDLD is a rare genetic corneal disease. Up to date, only one patient with GDLD was reported from China, with compound heterozygous mutation Q118X/Y184C [10]. Q118X is the most frequent mutation found in Japanese GDLD patients [4]. The previous Chinese GDLD case lived in northern China, whose ethnic relationship with Japanese was unclear. Our cases are from two unrelated consanguineous families in the southern and southeastern regions of China, and the mutations are found to be novel. Moreover, our patients have no ethnic relationship with the Japanese.

Apart from the mutations reported in Japan previously, molecular heterogeneity has also been observed in India, the United States, Europe, Tunisia [7], Turkey [8], and Vietnam [9]. The two novel mutations identified in this study demonstrate further mutational heterogeneity. Studies of the genetic basis of the corneal dystrophies have revealed that most reported cases of gelatinous corneal dystrophy are caused by amino acid substitutions within the *TACSTD2* gene, and most reported mutations have been missense and nonsense mutations. The two novel mutations identified in our current study are both homozygous frame-shift mutations, and the sites of the mutations are unique. These consisted of two insertions/deletions of a nucleotide, causing a shift in the translational reading frame and introducing a premature termination codon not far downstream of the mutation site. Similar mutations that have been reported previously are c.653delA [8], c.632delA, and c.520insC [4,13].

The 40 kDa *TACSTD2* protein contains an epidermal growth factor-like repeat, a thyroglobulin repeat, a transmembrane domain, and a phosphatidylinositol (PIP2)-binding site, harboring phosphorylatable serine and threonine residues near the COOH-terminus [4]. Mutation c.84insG in *TACSTD2* results in a predicted stop codon at nt280. The effect of this truncating mutation is loss of the thyroglobulin repeat, the transmembrane domain, and the PIP2-binding site. Mutation c.480delC is similar to c.653delA, c.632delA, and c.520insC, which result in structural removal of the transmembrane domain and the PIP2-binding site from the COOH-terminus of the protein.

At least 20 mutations of *TACSTD2* gene have been found thus far. Considerable phenotypic variation has been reported [2,7]. The phenotype-genotype correlation is not clear. It still needs to be determined if the mutational pattern is correlated with the clinical course, or may be a prognostic indicator. As in our study, both occurrences are of the typical mulberry type according to the classification proposed in Japan [2], whereas the clinical manifestation of proband A with mutation c.84insG was more severe than proband B with mutation c.480delC. Further investigation is needed to determine whether an earlier stop codon generated by mutation c.84insG could influence phenotype severity. More cases are needed to analyze the phenotype-genotype correlation.

The role of mutations in *TACSTD2* in the pathogenesis of GDLD is also not well understood. Protein expression analysis revealed perinuclear aggregation of the mutated, truncated protein, whereas the normal protein was distributed diffusely in the cytoplasm with a homogenous or fine granular pattern [4]. Further elucidation of the cellular and molecular aspects of function may provide insight into pathogenesis.

## References

[1] Fujiki K Nakayasu K Kanai A Corneal dystrophies in Japan. J Hum Genet 2001 46 431 5 11501939 10.1007/s100380170041

[2] Ide T Nishida K Maeda N Tsujikawa M Yamamoto S Watanabe H Tano Y A spectrum of clinical manifestations of gelatinous drop-like corneal dystrophy in japan. Am J Ophthalmol 2004 137 1081 4 15183793 10.1016/j.ajo.2004.01.048

[3] Santo RM Yamaguchi T Kanai A Okisaka S Nakajima A Clinical and histopathologic features of corneal dystrophies in Japan. Ophthalmology 1995 102 557 67 7724173 10.1016/s0161-6420(95)30982-7

[4] Tsujikawa M Kurahashi H Tanaka T Nishida K Shimomura Y Tano Y Nakamura Y Identification of the gene responsible for gelatinous drop-like corneal dystrophy. Nat Genet 1999 21 420 3 10192395 10.1038/7759

[5] Ripani E Sacchetti A Corda D Alberti S Human Trop-2 is a tumor-associated calcium signal transducer. Int J Cancer 1998 76 671 6 9610724 10.1002/(sici)1097-0215(19980529)76:5<671::aid-ijc10>3.0.co;2-7

[6] Taniguchi Y Tsujikawa M Hibino S Tsujikawa K Tanaka T Kiridoushi A Tano Y A novel missense mutation in a Japanese patient with gelatinous droplike corneal dystrophy. Am J Ophthalmol 2005 139 186 8 15652848 10.1016/j.ajo.2004.06.090

[7] Ren Z Lin PY Klintworth GK Iwata F Munier FL Schorderet DF El Matri L Theendakara V Basti S Reddy M Hejtmancik JF Allelic and locus heterogeneity in autosomal recessive gelatinous drop-like corneal dystrophy. Hum Genet 2002 110 568 77 12107443 10.1007/s00439-002-0729-z

[8] Markoff A Bogdanova N Uhlig CE Groppe M Horst J Kennerknecht I A novel TACSTD2 gene mutation in a Turkish family with a gelatinous drop-like corneal dystrophy. Mol Vis 2006 12 1473 6 http://www.molvis.org/molvis/v12/a166/17167402

[9] Ha NT Chau HM Cung le X, Thanh TK, Fujiki K, Murakami A, Kanai A. A novel mutation of M1S1 gene found in a Vietnamese patient with gelatinous droplike corneal dystrophy. Am J Ophthalmol 2003 135 390 3 12614764 10.1016/s0002-9394(02)01952-9

[10] Tian X Fujiki K Li Q Murakami A Xie P Kanai A Wang W Liu Z Compound heterozygous mutations of M1S1 gene in gelatinous droplike corneal dystrophy. Am J Ophthalmol 2004 137 567 9 15013888 10.1016/j.ajo.2003.08.008

[11] Grimberg J Nawoschik S Belluscio L McKee R Turck A Eisenberg A A simple and efficient non-organic procedure for the isolation of genomic DNA from blood. Nucleic Acids Res 1989 17 8390 2813076 10.1093/nar/17.20.8390 PMC334995

[12] Akhtar S Bron AJ Qin X Creer RC Guggenheim JA Meek KM Gelatinous drop-like corneal dystrophy in a child with developmental delay: clinicopathological features and exclusion of the M1S1 gene. Eye 2005 19 198 204 15254496 10.1038/sj.eye.6701453

[13] Tasa G Kals J Muru K Juronen E Piirsoo A Veromann S Janes S Mikelsaar AV Lang A A novel mutation in the M1S1 gene responsible for gelatinous droplike corneal dystrophy. Invest Ophthalmol Vis Sci 2001 42 2762 4 11687514

